# Predictors of Weight Reduction in a Multidisciplinary Community Program for Children with Overweight and Obesity: A Study from Emilia-Romagna, Italy

**DOI:** 10.3390/nu17183015

**Published:** 2025-09-20

**Authors:** Gianmarco Imperiali, Cecilia Acuti Martellucci, Marina Fridel, Giuseppe Diegoli, Maurizio Iaia, Giuliano Carrozzi, Petra Bechtold, Maria Elena Flacco, Lamberto Manzoli

**Affiliations:** 1 Department of Medical and Surgical Sciences, University of Bologna, 40126 Bologna, Italy; gianmarco.imperiali2@studio.unibo.it (G.I.); c.acutimartellucci@unibo.it (C.A.M.); 2 Public Health Department, Emilia-Romagna Region, 40127 Bologna, Italy; marina.fridel@regione.emilia-romagna.it (M.F.); giuseppe.diegoli@regione.emilia-romagna.it (G.D.); maurizio.iaia@icloud.com (M.I.); 3 Affiliation Epidemiology and Risk Communication Service Modena Local Health Authority (LHA), Emilia-Romagna Region, 41126 Modena, Italy; g.carrozzi@ausl.mo.it (G.C.); p.bechtold@ausl.mo.it (P.B.); 4 Department of Environmental and Prevention Sciences, University of Ferrara, 44121 Ferrara, Italy; mariaelena.flacco@unife.it

**Keywords:** child/adolescent, obesity, community-based, lifestyle weight-management program, Italy

## Abstract

**Background**: The worldwide prevalence of obesity in children and adolescents quadrupled in the past decades, becoming a public health priority. Following the recommendation by the Italian Minister of Health, the Emilia-Romagna Region started a community-based program aimed at reducing pediatric overweight through children and family behavioral counseling on nutrition and physical activity. **Methods**: Children with excess weight, aged 2–17 years, and without severe diseases were visited five times by a multidisciplinary team, who provided dietary advice, exercise plans, and psychosocial support, according to Italian guidelines. The outcomes were the median pre–post change in Δ_30_BMI (distance between children’s BMI and age- and sex-specific obesity threshold values) and the proportion of children who moved to a lower weight class. Logistic regression was used to identify potential predictors of weight improvement. **Results**: Up to March 2025, 1331 participants completed the follow-up. In total, 17.5% of the children showed an improvement in weight class, and 32.5% had a reduction of more than one unit of Δ_30_BMI. The program was significantly and substantially more effective among the children with obesity at baseline (overweight vs. obese children adjusted odds ratio—aOR—of weight class improvement: 0.28; *p* < 0.001), older than eight years (9–11 years vs. 2–8 years children aOR: 1.41; *p* < 0.05), who adhered to breakfast recommendations (aOR: 1.60; *p* < 0.01) and had no obese parents (≥1 vs. 0 obese parents aOR: 0.62; *p* < 0.05). **Conclusions**: The multidisciplinary model was associated with an overall positive impact on the weight status of the enrolled children. Given the varying response, however, in order to maximize cost-effectiveness, future programs could be reserved for children with obesity, older than eight years. Further randomized research is needed to investigate the efficacy of this intervention in different settings and on late clinical endpoints.

## 1. Introduction

According to the World Health Organization (WHO), the estimated worldwide prevalence of obesity in children and adolescents increased from 2% in 1990 to 8% in 2022 [1]. This trend raises important concerns for public health, as children with obesity are more likely to continue experiencing excess weight into adulthood [2], thus increasing their risk of chronic diseases [3,4,5], whereas obese children who become normal-weight adults show rates of cardiovascular diseases that are similar to their non-obese counterparts [6].

In order to reduce the expansion of childhood obesity, the WHO recommended the implementation of obesity management programs for children and adolescents [7,8]. In the last decades, a number of these interventions have been implemented and evaluated, either through randomized controlled trials (RCTs) [9] or observational studies [10], and several studies have suggested that community-based prevention programs, which typically include multidisciplinary teams and family involvement, could be effective in fostering lifestyle changes [11,12,13]. These programs emphasize the promotion of a balanced diet, regular physical activity, and the reduction of sedentary behaviors, addressing the environmental and behavioral determinants of overweight and obesity [11].

In the midst of often contrasting evidence, as the effectiveness of these programs appears to vary widely in relation to study setting, duration, and inclusion of nutritional, physical activity, or behavioral components [10], real-world data which identify responding subgroups and which would aid in order to optimize public policy are lacking.

A recent report estimated that approximately 19.0% and 9.8% of Italian 9-year-old children were classified as overweight or obese, respectively, highlighting the need of targeted population-based interventions [14]. The Emilia-Romagna Region, located in northern Italy, is not free from the threat of childhood overweight [15], although the figures are slightly lower compared to the national mean: 18.6% and 7.1% of overweight and obese children, respectively [16,17].

In 2017, the Emilia-Romagna Region implemented a community-based, multifaceted program, called “Bimbi in forma”, which has been described in detail in the WHO Europe report “Mapping the health system response to childhood obesity in the WHO European Region: an overview and country perspectives” [7] and in a dedicated page of the Italian National Institute of Health website [18], with the aim of improving the lifestyle of children with excess weight through multidisciplinary educational and family-centered interventions. In this study, we report a pre–post evaluation of the impact of the program, from its beginning in December 2017 up to March 2025.

## 2. Materials and Methods

### 2.1. Study Intervention

Starting from December 2017, the primary care pediatricians (PCPs) operating in the region were requested to refer all children aged between 2 and 17 years with excess weight (exceeding the International Obesity Task Force—IOTF—BMI threshold corresponding to 25 kg/m^2^ for adults), who did not respond to their standard care, for enrolment in the program. Children with severe diseases or those who needed pharmacological treatments or bariatric surgery were directly referred to hospital-based tertiary care and were not included in the program. The parents of the eligible children, who agreed to participate with their offspring, were requested to sign an informed consent form. Once they entered the program, all children were followed by a multidisciplinary team composed of one pediatrician, a dietitian, a sports medicine specialist, a kinesiologist, and a psychologist. The team performed a comprehensive evaluation and, based on children’s age and weight, provided dietary advice, exercise plans, and psychosocial support, according to the Italian guidelines on pediatric excess weight management [19,20]. This standardized approach was based on the one listed by the WHO among the best practices for tackling childhood obesity [7,18]. The program required the children to be evaluated five times within a minimum of six up to a maximum of 24 months. One multidisciplinary team was assembled in each local health authority (LHA) of the region, and all visits were provided free of charge.

The standardization of procedures across the different LHAs was ensured through at least three yearly meetings with all the members of all the multidisciplinary teams. In cases when a healthcare professional wished to join a team, they had to complete a 12 h online course and to participate in at least one meeting with the territorial multidisciplinary team.

The study protocol was approved by the internal board of the Emilia-Romagna Region, which did not request a formal evaluation from the Regional Ethics Committee, and the analyses were conducted using only secondary, already collected data.

### 2.2. Outcomes

Demographic (gender and age), anthropometric (height and weight), and lifestyle (physical activity levels, sedentary behaviors, daily portions of fruit and vegetables, sugared beverages consumption, breakfast habits) information was collected at the first and fifth visit by the multidisciplinary teams. At both assessments, the weight class of each child was assigned according to the cut-offs by Cole et al. [21]. Drawing from past methodological research [22,23,24,25,26] and in an attempt to maximize the standardization of results, Δ_30_BMI was computed as the difference between children’s BMI (standardized for age and sex using the International Obesity Task Force (IOTF) method) and the age- and sex-specific BMI values corresponding to an adult BMI of 30 kg/m^2^. Δ_30_BMI values should be clinically interpreted as follows:

- the value 0 is given to a child with a weight status equivalent to an adult with BMI 30;
- a value below 0 means that the child has a weight status similar to an adult with BMI lower than 30 and vice versa (Δ_30_BMI > 0 equals an adult BMI > 30);
- with a similar logic, (a) Δ_30_BMI values comprised between −5 and −0.1 are equivalent to adult BMI values ranging from 25 to 29.9 (overweight), and a Δ_30_BMI value lower than −5 indicates an adult BMI < 25 (normal weight); (b) Δ_30_BMI values between +0.1 and +5 are equivalent to adult BMI values ranging from 30.1 to 35 (obesity), and a Δ_30_BMI value higher than +5 indicates an adult BMI > 35 (severe obesity).

Therefore, children were assigned to the overweight, obesity, and severe obesity classes using the following thresholds, respectively: −5 ≤ Δ_30_BMI < 0; 0 ≤ Δ_30_BMI < 5; and Δ_30_BMI ≥ 5.

The primary outcome of the study was the mean change in Δ_30_BMI after six months of follow-up (difference between the first and fifth visit). The secondary outcome was the proportion of children who moved to a lower weight class (e.g., from obese to overweight).

Lifestyle data were collected using a semi-standardized questionnaire based on the National Framework for the Surveillance of Lifestyle Habits of Children [27]. Using the thresholds for food consumption/physical activity/screen time recommended by the international guidelines [28,29,30,31,32] (reported in Table S1), the responses on lifestyles habits were dichotomized as “adherence” or “non-adherence” to the guidelines. Breakfast habits were classified as “no breakfast” when no food was consumed in the morning after waking up, “adequate” when it consisted of a complete meal with at least one source of carbohydrates and one of proteins, and “inadequate” when breakfast was not compliant with the former definition, as reported in the National Framework for the Surveillance of Lifestyle Habits of Children [27]. Self-reported weight status and educational levels of parents were also collected.

### 2.3. Data Analysis

The children who did not complete the fifth visit within 24 months of the first visit were considered dropouts. However, those who did not complete the fifth visit but underwent the first visit within the 24 months before the end of the follow-up (11 March 2025) could not be considered dropouts and were excluded from the analyses. For the final sample with a complete follow-up, descriptive statistics were used to summarize the main characteristics of the sample at the first and fifth visit. The Wilcoxon signed-rank test for paired data was used to evaluate changes in continuous variables distributions from the start to the end of the follow-up, while the McNemar test was used to evaluate pre–post differences between dichotomous variables.

The potential independent predictors of improvement in weight were evaluated using multivariable random-effect logistic regression, using LHU as the cluster variable. Two separate models were built using two outcomes: (1) a decrease in Δ_30_BMI larger than one unit (selected in view of the established linear association between BMI and serological markers of cardiovascular disease [33,34]); (2) a change from a higher to a lower weight class (e.g., from obesity to overweight). Both models were adjusted for age, gender, and improvement in lifestyle habits. Mother’s educational level and parents’ weight status had, respectively, 90 and 104 missing observations and were included in separate models, with all other covariates equal. None of the other covariate estimates changed substantially after this inclusion. Thus, we showed the results of the final models without missing data (excluding the two covariates) and opted not to use missing data imputation techniques. The results of the logistic regression analyses were reported as odds ratios (ORs) and 95% confidence intervals (CIs).

In order to assess the robustness of the results to attrition, we predicted both BMI outcomes for the dropouts using multiple imputation of missing values, with bootstrap estimation and 10 repetitions. The prediction matrix was based upon gender, age, and weight class at baseline [35,36].

All tests were two-sided, with the significance level set at *p* < 0.05. All analyses were carried out using Stata, version 13.1 (Stata Corp., College Station, TX, USA, 2013).

## 3. Results

The characteristics of the sample, both at baseline and at the end of follow-up, are listed in Table 1. Of the 1792 children/adolescents included in the program up to March 2023, 1331 completed the fifth visit within two years and comprised the final sample, while 461 did not complete the program (25.7%). The mean age of the final sample was 10.3 years (SD: 2.7); 53.0% were males, and 51.0% had at least one parent with obesity. The differences between those who completed the follow-up and the dropouts were limited, with the latter being slightly older (10.7 years) and more frequently severely obese (43.4%). Most of the fifth visits (68.3%) were performed within a year from the first visit (average time: 9.5 months), but the average contact time varied widely by LHU, from a minimum of 5 ± 2 months (Reggio-Emilia) up to 15 ± 4 (Modena).

### 3.1. Lifestyle Habits

At the first visit, most of the children/adolescents had inadequate lifestyle habits, with the sole exception of fruit and vegetables consumption, as 64.4% of the participants consumed ≥4 portions per day (Table 1). Especially poor was the attitude towards physical activity, which was adequate only for 7.2% of the participants. At the fifth visit, all lifestyle habits significantly improved (all *p* < 0.001), with 66.4% of the children consuming an adequate breakfast, 71.4% drinking <1 sugared drink per week, 52.3% spending ≤2 h of daily sedentary screen time, 85.5% consuming ≥4 portions of fruit and vegetables per day, and 81.0% spending an adequate amount of time doing physical activity.

### 3.2. Overweight/Obesity

**Figure 1:**
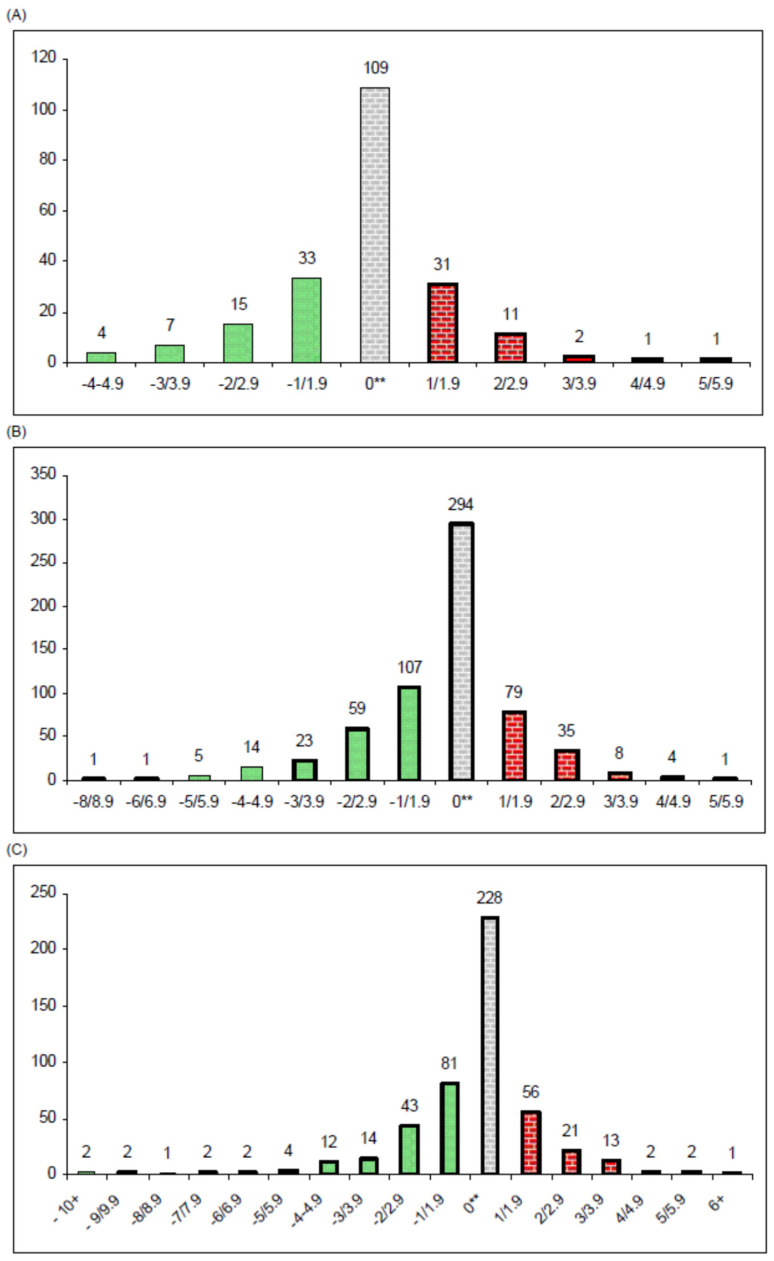
Histogram describing the distribution of the changes in Δ_30_BMI values between the fifth and the first visit **, stratified by baseline weight class: (**A**) overweight children only (*n* = 214); (**B**) children with obesity only (*n* = 631); (**C**) children with severe obesity only (*n* = 486). ** Negative values indicate an improvement in weight status (decrease in Δ_30_BMI) at the fifth visit, while positive values indicate a worsening in weight status (increase in Δ_30_BMI) at the fifth visit. Δ_30_BMI refers to the difference between children’s body mass index (BMI) and the age- and sex- specific BMI value corresponding to an adult BMI of 30 kg/m^2^.

At baseline, 83.9% of the children/adolescents were classified as obese (36.5% severely obese), with a median Δ_30_BMI of 2.8 (IQR: 4.4; Table 1). At the end of the follow-up, the prevalence of severe obesity remained unaltered, but both the median Δ_30_BMI and the proportion of obesity decreased significantly (from 47.4% to 40.4%); overweight increased, and 17 participants achieved normal weight (1.3%). Approximately one fifth of the sample (*n* = 268) gained weight (Figure 1), but 32.5% of the participating children/adolescents reduced their Δ_30_BMI by more than one unit, and 17.5% (*n* = 233) moved from a heavier to a lighter weight class (e.g., from obese to overweight; Table 2; Figure 1).

### 3.3. Predictors of Weight Improvement

Several factors were significantly associated with at least one weight improvement outcome, either at univariate or multivariable analysis (Table 2 and Table 3). First, a few predictors exist that were likely directly causing the outcome: indeed, the likelihood of a reduction of at least one unit of Δ_30_BMI and/or of an improvement in weight class was significantly higher among the children/adolescents who, at the end of follow-up, were following the recommendations for each of the five selected lifestyle habits: breakfast, fruit, vegetables and sugared beverages consumption, physical activity, and screen time.

**Figure 2:**
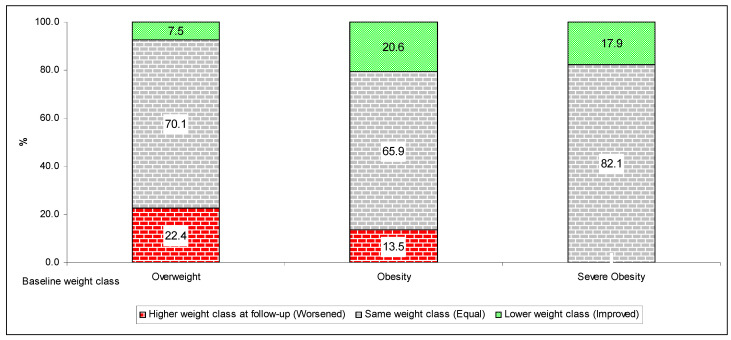
Histogram describing the distribution of the changes in weight class between the first and fifth visit. A higher weight class indicates an overweight child at baseline (or obese), who was then classified obese (or severely obese) at the fifth visit. A lower weight class indicates a child who was overweight at baseline and normal at the fifth visit; obese at baseline and normal or overweight at the fifth visit; or severely obese at baseline and normal, overweight, or obese at the fifth visit.

Also, as compared to the younger children (2–8 years), the probability of success was much higher for the older children (9–11 years) and especially for the adolescents (12–17 years), who showed an adjusted odds ratio (OR) of Δ_30_BMI improvement of 2.21 (95% confidence interval—CI: 1.54–3.17; Table 3). The other significant independent predictors of program effectiveness were baseline children’s and parents’ weight class and time from the first and the fifth visit. At multivariable analyses (Table 3), the participants who waited 12 or more months after the first visit, as well as those with at least one obese parent, showed a significantly lower likelihood of weight improvement. Similarly, the children/adolescents who were overweight at baseline, as compared to those with obesity, were significantly less likely to reduce Δ_30_BMI by one unit or more and especially to decrease in weight class (adjusted OR: 0.28; 95% CI: 0.16–0.48). As shown in Figure 2, among the participants who were overweight at baseline, those who reduced their weight class during the follow-up were three times less than those who passed to a higher class (7.5% vs. 22.4%, respectively). In contrast, among the children/adolescents with obesity, 20.6% improved and 13.5% worsened their weight class during the follow-up.

No impact on the program effectiveness was observed for gender and mother’s educational status (all multivariable *p* > 0.1).

## 4. Discussion

This WHO-derived, community-based, multidisciplinary, lifestyle educational program targeted at children and adolescents with excess weight showed positive results, as one in three participants reported a clinically meaningful BMI reduction. Importantly, the effectiveness varied largely by baseline weight class, as the children/adolescents with obesity responded much better than their overweight peers. Also, the program was significantly and substantially more effective among the participants older than eight years, who adhered to breakfast recommendations, with no obese parents.

As mentioned, the overall effectiveness of the program was good, with almost one third of the participants showing a clinically meaningful BMI reduction, and one sixth moved to at least one weight class lower. The literature on the topic is wide, with several meta-analyses of RCTs and/or observational studies that have explored the impact of lifestyle programs on children and adolescents with overweight or obesity and results ranging from no detectable effects [37,38] to small positive effects [39,40,41,42,43,44,45,46], especially for strategies including exercise and/or family-based approaches [47,48]. The two largest meta-analyses of RCTs to date suggest a moderate overall effectiveness [49], especially for after-school programs and community-based interventions [9]. On average, when looking at observational evidence, for various reasons not limited to typical methodological biases [50,51,52,53], the effectiveness was higher [54,55], as it was in one recent pre–post Italian evaluation similar to the one described in the present report [56]. However, two similar studies from the UK found comparable BMI reductions after one year [57,58], while a further study from Norway did not detect any clinically meaningful reductions in BMI after almost three years of follow-up, underscoring the difficulty to maintain benefits in the longer term [59].

A significantly greater effectiveness of the program was observed among the participants who were obese or severely obese at baseline compared to the overweight ones whose overall response was poor. The same finding emerged from two meta-analyses [40,43] and has important policy implications. In fact, in the many contexts with limited resources or simply to maximize the intervention cost-effectiveness, priority should be given to individuals with more severe excess weight, and, provided that this trend is confirmed through randomized studies, future programs should be reserved for children or adolescents with obesity.

Another important finding was the higher response observed in the participants aged ≥8 years, who were more likely to improve. This finding is in line with a large meta-analysis of RCTs [49] and some [60], but not all [61,62], observational studies. Clearly, the implications for health policies are similar to the above finding on weight class: should the results be confirmed, the ensuing recommendation, aimed at the best allocation of resources, would be to direct educational programs to children who are obese and aged eight years or more. At the same time, when identifying the ideal target age-group, policy makers should keep in mind the possibility of adolescents being more prone to withdraw from interventions. Indeed, in the present investigation, as well as in another Italian study [63], higher rates of dropout were observed among older children. On the contrary, further international evaluations report no or negative associations [64,65,66], suggesting once more that the impact of weight reduction strategies is highly context-dependent and that target populations should be carefully defined in view of the available evidence and resources.

In this study, lifestyle habits were classified according to the adherence to international guidelines [28] in order to evaluate whether achieving the recommended thresholds was associated with significant improvements in BMI. As mentioned in the results, it should be kept in mind that most of the investigated lifestyle habits were found to directly influence BMI [28,67], especially physical activity [68,69], and indeed, in the present study, the BMI reduction was strongly and positively associated with conforming to physical activity guidelines [28,29]. Specifically, over three quarters of the sample transitioned to engaging in sufficient exercise during the program, although this change was not matched by an equivalent improvement in the weight outcomes, as only one third obtained a significant BMI reduction. This is to be expected given the limited time-frame of the intervention, which was below one year for the majority of participants [60,61], but it could also be due to an undetected increase in energy intake [70], although its link to an increase in energy expenditure is uncertain for children/adolescents [71,72]. Concurrently, the impact of social desirability bias might be relevant, whereupon self-reports of physical activity are likely overestimated [73,74], even more so by those undergoing an active intervention [75].

More than exercising, consuming an adequate breakfast as well as having non-obese parents were strongly associated with BMI improvements. Both findings are consistent with the literature: several studies showed an association between skipping breakfast and childhood obesity [76,77,78,79], and other analyses suggested that both genetics and the social environment are likely to contribute as mediators to the impact of weight interventions [80,81,82]. Unexpectedly, however, adhering to the recommendations on screen time [30], fruit and vegetables [31], and sugared beverages [32] consumption had no impact on weight reductions. This finding contrasts with a substantial body of research [83,84,85] and could merely reflect the uncertainties around the thresholds [86] and/or various information biases [87]. Also, the wide confidence intervals around the predictor estimates suggest that this study might have been underpowered to detect small effect sizes. Certainly, additional data are needed to refine the recommended thresholds for key lifestyle behaviors.

It should be noted that community-based lifestyle interventions have shown the potential to improve not only the physical health of participants but also their psychological health [88] and to have spillover effects on the family members [89]. Indeed, excess weight is known to affect disproportionately groups with lower socioeconomic status and limited health literacy [90,91,92], while also predicting delays in seeking care [93] and higher healthcare utilization [94]. Consequently, lifestyle interventions involving the whole family, such as the one promoted by the Emilia-Romagna Region, could positively impact not only the single participant but also those connected to them [95]. In a recent assessment, the most commonly reported positive spillover was for nutrition-related outcomes among siblings and parents [89]. However, although the described program was targeted not only to the single child but to the whole family, to verify potential improvements in the psychological health of participants or in the overall health of other family members was beyond the scope of this study and warrants future investigations.

### 4.1. Strengths and Limitations

The strengths of this study include the use of WHO guideline-based recommendations and standardized protocols to measure outcomes and collect relevant variables, thus enhancing methodological consistency [21]. Indeed, the classification of lifestyle habits through guideline-derived thresholds allowed the assessment of the impact of these recommendations on weight reduction [19]. Importantly, when the missing follow-up BMI values of the dropouts were imputed using multiple imputation, the adjusted odds ratios did not substantially differ (Table S1), suggesting that the results were robust in spite of a high attrition rate. The study has also limitations that must be taken into account when interpreting the results. First, as this work analyzes an ongoing, long-standing public health program, it was not possible to test the intervention’s efficacy against a control group. Second, as mentioned, although the healthcare professionals directly assessed anthropometric measures during the visits, lifestyle habits were collected using standardized self-reporting tools. Indeed, to enhance acceptability by children and families, the present study did not include any objective measures (e.g., dietary logs or accelerometers for physical activity), thus introducing the risk of recall and social desirability biases [87]. In specific, the youth involved in weight reduction interventions are more likely to report improved habits compared to their peers who are not targeted by any intervention [75], suggesting that the observed improvements are likely overestimated and that their association with weight outcomes could, instead, be underestimated in the event that children with no weight reductions are more prone to lie about their habits. Third, the resources allocated for the program did not allow for the collection of any outcome measure aside from BMI. While remaining the most practical measure, BMI has been criticized by several authors, primarily due to its inability to discriminate between fat and fat-free mass [26], and additional clinical endpoints would have painted a more complete picture of the program’s impact and shortcomings. Finally, the results refer to the first ten months of follow-up, and a longer-term evaluation is ongoing to verify whether the program’s impact substantially varied over time. Indeed, as previously mentioned, the duration of these interventions appears to play a crucial role: several authors noted that longer durations lead to better weight outcomes [43,59,96], although there is no evidence that the “intensity” (i.e., contact time) should also be increased [97].

### 4.2. Future Research Directions

Primarily, further investigations are necessary to understand the observed sizeable dropout rate: a more detailed assessment of participant characteristics, such as socio-demographic and motivational factors [63], would be beneficial for the retention of future programs. Moreover, either in novel studies or in sub-samples of the present one, longitudinal evaluations should assess clinical endpoints such as metabolic parameters, psychosocial conditions, and quality-of-life measures.

Also, although the present findings suggest that older and heavier children/adolescents are more likely to improve, a further exploration of potential underlying behavioral or physiological mechanisms should be conducted. This could be carried out, for example, through the longitudinal tracking of participants, bearing in mind that before any large-scale reallocations are made in order to restrict the program to specific subgroups, RCTs and cost-effectiveness studies are definitely needed.

More in general, as mentioned above, long-term evaluations of the subjects included in the program are strongly warranted to check whether the observed benefits are maintained after the conclusion of the intervention. Lastly, randomized studies, possibly even stepped wedge cluster randomized trials [98] based on objective behavior measures should be conducted to clearly define the efficacy of this program, and economic evaluations should also be performed to compare costs with other pediatric interventions, therefore ensuring the equitable allocation of resources while maximizing benefits for children and adolescents.

## 5. Conclusions

In conclusion, this study suggest that the multidisciplinary model implemented in the Emilia-Romagna Region was associated with an overall positive impact on the weight status of the enrolled children. However, the program showed significantly and substantially better results among the children who were obese at baseline and/or older than eight years. An evaluation of the long-term impact of the model is ongoing, and further randomized research is needed to investigate the efficacy of this intervention in different settings and on late clinical endpoints.

## Figures and Tables

**Table 1:** Sample characteristics, lifestyle habits, and weight at the start and the end of follow-up.

	1st Visit Overall Sample (*n* = 1792)	1st Visit Dropout (*n* = 461)	*p* ^A^	1st Visit Completed Follow-Up (*n* = 1331)	5th Visit Completed Follow-Up (*n* = 1331)	*p* ^B^
Characteristics	% (*n*)	% (*n*)		% (*n*)	% (*n*)	
*Gender*			0.244			
- Female	46.1 (827)	43.8 (202)		47.0 (625)	47.0 (625)	--
- Male	53.9 (965)	56.2 (259)		53.0 (706)	53.0 (706)	--
Mean age in years (SD)	10.4 (2.7)	10.7 (2.8)	0.001	10.3 (2.7)	11.0 (2.8)	<0.001
*Baseline age class in years*						
- 2–8	29.6 (530)	23.6 (109)	0.001	31.6 (421)	31.6 (421)	--
- 9–11	39.5 (707)	40.6 (187)	0.570	39.1 (520)	39.1 (520)	--
- 12–17	31.0 (555)	35.8 (165)	0.001	29.3 (390)	29.3 (390)	--
*Mother’s educational level* ^C^			0.114			
- High school degree or lower	81.0 (1297)	83.9 (302)		80.2 (995)	80.2 (995)	--
- University degree	19.0 (304)	16.1 (58)		19.8 (246)	19.8 (246)	--
*Parents’ weight status* ^D^						
- Either underweight or normal weight	10.5 (170)	10.4 (40)	0.904	10.6 (130)	10.6 (130)	--
- At least one overweight	37.4 (603)	34.2 (132)	0.108	38.4 (471)	38.4 (471)	--
- At least one obese	55.4 (840)	55.4 (214)	0.103	51.0 (626)	51.0 (626)	--
*Adequate breakfast*						
- No breakfast	21.2 (380)	27.1 (125)	<0.001	19.2 (255)	8.5 (113)	<0.001
- Inadequate	50.9 (913)	45.3 (209)	0.005	52.9 (704)	25.1 (334)	<0.001
- Adequate ^E^	27.9 (499)	27.6 (127)	0.901	27.9 (372)	66.4 (884)	<0.001
*Fruit and vegetables*			0.501			
- Less than 4 portions per day	36.0 (645)	37.1 (171)		35.6 (474)	14.5 (193)	<0.001
- 4+ portions per day ^E^	64.0 (1147)	62.9 (290)		64.4 (857)	85.5 (1138)	<0.001
*Sugared beverages*			0.611			
- 1+ portions per week	60.8 (1090)	61.8 (285)		60.5 (805)	28.6 (380)	<0.001
- Less than one portion per week ^E^	39.2 (702)	38.2 (176)		39.5 (526)	71.4 (951)	<0.001
*Physical activity*			0.069			
- Inadequate	93.4 (1674)	95.2 (439)		92.8 (1235)	19.0 (251)	<0.001
- Adequate ^E^	6.6 (118)	4.8 (22)		7.2 (96)	81.0 (1070)	<0.001
*Screen time*			0.150			
- >2 h	58.8 (1053)	61.6 (284)		57.8 (769)	47.7 (635)	<0.001
- ≤2 h ^E^	41.2 (739)	38.4 (177)		42.2 (562)	52.3 (696)	<0.001
*Weight class*						
- Normal weight	--			--	1.3 (17)	--
- Overweight	15.3 (275)	13.2 (61)	0.14	16.1 (214)	21.8 (290)	<0.001
- Obesity	46.4 (831)	43.4 (200)	0.14	47.4 (631)	40.4 (538)	<0.001
- Severe obesity	38.3 (686)	43.4 (200)	0.009	36.5 (486)	36.5 (486)	0.96
Median Δ_30_BMI ^F^ (IQR)	2.9 (4.5)	3.6 (5.2)	<0.001	2.8 (4.4)	2.3 (5.1)	<0.001
Mean time from 1st to 5th visit in months (SD)	--	--	--	9.6 (5.5)	9.6 (5.5)	--
Months from 1st to 5th visit (classes)						
- 3–6	--	--	--	31.0 (413)	31.0 (413)	--
- 7–12	--	--	--	37.3 (496)	37.3 (496)	--
- 12–24	--	--	--	31.7 (422)	31.7 (422)	--

SD = standard deviation. IQR = interquartile range. BMI = body mass index. ^A^ The difference between the dropouts and the group that completed the fifth visit follow-up were evaluated using chi-squared test for categorical variables and *t*-test for continuous ones. ^B^ The difference between the first and fifth visit was evaluated using a paired *t*-test for continuous variables and McNemar test for categorical ones. ^C^ 191 missing values (90 among those who completed the follow-up). ^D^ 179 missing values (104 among those who completed the follow-up). ^E^ Adherence to international guidelines achieved. ^F^ Δ_30_BMI refers to the difference between children’s BMI and the age- and sex-specific BMI value corresponding to an adult BMI of 30 kg/m^2^. Therefore, a positive Δ_30_BMI indicates that the child’s BMI is above the obesity threshold, and negative values indicate it is below.

**Table 2:** Percentage of participants with improvement in weight outcomes overall and stratified by demographic, anthropometric, and lifestyle variables.

	Improvement in Δ_30_BMI ^A^		Improvement in Weight Class	
	% (*n*/*N*)	*p* *	% (*n*/*N*)	*p* *
Overall	32.5 (432/1331)	--	17.5 (233/965)	--
*By baseline weight class*				^1, 2, 3^
- Overweight	27.6 (59/214)		7.5 (16/214)	
- Obesity	33.3 (210/631)		20.6 (130/631)	
- Severe obesity	33.5 (463/489)		17.9 (87/486)	
*By baseline age class in years*		^1, 2, 3^		
- 2–8	26.1 (110/421)		14.7 (62/421)	
- 9–11	32.1 (167/520)		17.9 (93/520)	
- 12–17	39.7 (155/390)		20.0 (78/390)	
*By gender*		0.104		0.551
- Female	30.2 (189/625)		16.6 (104/625)	
- Male	34.4 (243/706)		18.3 (129/706)	
*By adequate breakfast at the 5th visit* ^B^		^3^		^1, 3^
- No breakfast	27.4 (31/113)		15.0 (17/113)	
- Inadequate	25.5 (85/334)		12.6 (42/334)	
- Adequate	35.8 (316/884)		19.7 (174/884)	
*By fruit and vegetables at the 5th visit* ^B^		0.001		0.112
- Less than 4 portions per day	22.3 (43/193)		13.5 (26/193)	
- 4+ portions per day	34.2 (389/1138)		18.2 (207/1138)	
*By sugared beverages at the 5th visit* ^B^		<0.001		0.069
- 1+ portions per week	25.3 (96/380)		14.5 (55/380)	
- Less than one portion per week	35.3 (336/951)		18.7 (178/951)	
*By physical activity at the 5th visit* ^B^		0.006		0.043
- Inadequate	30.7 (332/1080)		16.5 (178/1080)	
- Adequate	39.8 (100/251)		21.9 (55/251)	
*By screen time at the 5th visit* ^B^		0.489		0.314
- >2 h	33.4 (212/635)		16.4 (104/635)	
- ≤2 h	31.6 (220/696)		18.5 (129/696)	
*By mother’s educational level*		0.308		0.150
- High school degree or lower	32.0 (318/995)		16.8 (167/995)	
- University degree	35.4 (87/246)		20.7 (51/246)	
*By parents’ weight status*				^1, 2, 3^
- Either underweight or normal weight	39.2 (51/130)		23.9 (31/130)	
- At least one overweight	32.7 (154/471)		18.7 (88/471)	
- At least one obese	30.7 (192/626)		14.5 (91/626)	

BMI = body mass index. Improvement in Δ_30_BMI is defined as a post–pre decrease in Δ_30_BMI >= −1. Improvement in weight class is defined as a change from a higher weight class to a lower one (e.g., from obese to overweight). ^A^ Δ_30_BMI refers to the difference between children’s BMI and the age- and sex-specific BMI value corresponding to an adult BMI of 30 kg/m^2^. ^B^ Adherence to recommended thresholds of international guidelines on lifestyle habits. * Chi-squared test: ^1^ *p* < 0.05 of the difference between overweight children vs. children with obesity (both at baseline); ^2^ *p* < 0.05 of the difference between overweight children vs. children with severe obesity (both at baseline); ^3^ *p* < 0.05 of the difference between children with obesity vs. children with severe obesity (both at baseline).

**Table 3:** Adjusted odds ratios, 95% confidence interval, and *p*-values relative to predictors of improvement in weight outcomes.

	Improvement in Δ_30_BMI ^A^		Improvement in Weight Class ^A^	
Predictors	OR (95% CI)	*p* *	OR (95% CI)	*p* *
*Gender*				
- Female	1 (Ref. cat.)	--	1 (Ref. cat.)	--
- Male	1.19 (0.93–1.51)	0.165	1.08 (0.80–1.45)	0.614
*Age class in years*				
- 2–8	1 (Ref. cat.)	--	1 (Ref. cat.)	--
- 9–11	1.41 (1.03–1.94)	0.031	1.46 (0.99–2.14)	0.055
- 12–17	2.21 (1.54–3.17)	<0.001	1.99 (1.28–3.11)	0.002
*Weight class at the 1st visit*				
- Obesity	1 (Ref. cat.)	--	1 (Ref. cat.)	--
- Overweight	0.69 (0.48–0.98)	0.040	0.28 (0.16–0.48)	<0.001
- Severe obesity	1.22 (0.93–1.60)	0.146	0.99 (0.72–1.37)	0.966
*Adequate breakfast at the 5th visit*				
- None or inadequate	1 (Ref. cat.)	--	1 (Ref. cat.)	--
- Adequate	1.60 (1.19–2.15)	0.002	1.63 (1.14–2.35)	0.008
*Fruit and vegetables at the 5th visit*				
- Less than 4 portions per day	1 (Ref. cat.)	--	1 (Ref. cat.)	--
- 4+ portions per day ^B^	1.68 (1.19–2.15)	0.002	1.27 (0.79–2.03)	0.318
*Sugared beverages at the 5th visit*				
- 1+ portions per week	1 (Ref. cat.)	--	1 (Ref. cat.)	--
- <1 portion per week ^B^	1.48 (1.11–1.97)	0.008	1.20 (0.84–1.71)	0.316
*Physical activity at the 5th visit*				
- Inadequate	1 (Ref. cat.)	--	1 (Ref. cat.)	--
- Adequate	1.49 (1.09–2.04)	0.013	1.32 (0.91–1.92)	0.144
*Screen time at the 5th visit*				
- >2 h	1 (Ref. cat.)	--	1 (Ref. cat.)	--
- ≤2 h ^B^	1.08 (0.83–1.42)	0.566	1.33 (0.96–1.85)	0.086
*Mother’s educational level*				
- University degree	1 (Ref. cat.)	--	1 (Ref. cat.)	--
- High school degree or lower	1.14 (0.84–1.55)	0.411	1.29 (0.90–1.85)	0.172
*Parents’ weight status*				
- Either underweight or normal weight	1 (Ref. cat.)	--	1 (Ref. cat.)	--
- At least one overweight	0.71 (0.47–1.08)	0.112	0.68 (0.42–1.10)	0.118
- At least one obese	0.62 (0.41–0.94)	0.025	0.46 (0.28–0.75)	0.002
Time from 1st to 5th visit, months				
- 3–6	1 (Ref. cat.)	--	1 (Ref. cat.)	--
- 7–12	0.92 (0.68–1.25)	0.582	0.78 (0.53–1.14)	0.193
-12–24	0.54 (0.36–0.81)	0.003	0.85 (0.51–1.42)	0.536

BMI = body mass index. Improvement in Δ_30_BMI is defined as a post–pre decrease in Δ_30_BMI >= −1. OR = odds ratio. CI = confidence interval. Ref. cat. = reference category. ^A^ Δ_30_BMI refers to the difference between children’s BMI and the age- and sex-specific BMI value corresponding to an adult BMI of 30 kg/m^2^. Improvement in Δ_30_BMI is defined as a pre–post decrease in Δ_30_BMI > 1. Improvement in weight class is defined as a change from a higher weight class to a lower one. * Results obtained by two random-effect logistic regression models, using LHU as the cluster variable: the first predicting the improvement in Δ_30_BMI and the second predicting the improvement in weight class. Both models included 1331 children and were not adjusted for mother’s educational level and parents’ weight status, which were included into separate models (with all other covariates equal) because of 90 and 104 missing values, respectively. None of the other covariate estimates changed substantially after the inclusion of the mother’s educational level and parents’ weight status. ^B^ Adherence to recommended thresholds of international guidelines on lifestyle habits.

## Data Availability

The raw data supporting the conclusions of this article will be made available by the authors on request.

## Supplementary Materials

The following supporting information can be downloaded at https://www.mdpi.com/article/10.3390/nu17183015/s1. Table S1: Adjusted odds ratios, 95% confidence interval, and *p*-values relative to predictors of improvement in weight outcomes in a sample including also the 461 dropouts, whose missing data were estimated using multiple imputation.
