# Supplementary material for: Predictors of Weight Reduction in a Multidisciplinary Community Program for Children with Overweight and Obesity: A Study from Emilia-Romagna, Italy
Source: Nutrients. 2025 Sep 20;17(18):3015. doi: 10.3390/nu17183015 (PMC12473044; doi:10.3390/nu17183015)
Supplement: Supplementary file 1 [file nutrients-17-03015-s001.zip › nutrients-3885870-supplementary.pdf]

Table S1. Adjusted odds ratios, 95% confidence interval and p-values relative to predictors of improvement in weight outcomes, in a sample including also the 461 dropouts, whose missing data were estimated using multiple imputation.

	Improvement in Δ30BMI [A]		Improvement in weight class [A]	
**Predictors**	**OR (95% CI)**	**p***	**OR (95% CI)**	**p***
Gender				
- Female	1 (Ref. cat.)	--	1 (Ref. cat.)	--
- Male	1.17 (0.95–1.44)	0.139	1.04 (0.79–1.36)	0.794
Age class in years				
- 2-8	1 (Ref. cat.)	--	1 (Ref. cat.)	--
- 9-11	1.59 (1.20–2.09)	0.001	1.39 (0.97–1.99)	0.075
- 12-17	2.45 (1.82–3.30)	<0.001	1.73 (1.17–2.56)	0.006
Weight class at the 1st visit				
- Obesity	1 (Ref. cat.)	--	1 (Ref. cat.)	--
- Overweight	0.75 (0.55–1.03)	0.072	0.29 (0.17–0.49)	<0.001
- Severe obesity	1.24 (0.99–1.56)	0.064	0.90 (0.67–1.19)	0.451
Mother's educational level				
- University degree	1 (Ref. cat.)	--	1 (Ref. cat.)	--
- High school degree or lower	1.19 (0.90–1.57)	0.214	1.37 (0.95–1.98)	0.095
Parents weight status				
- Both underweight or normal weight	1 (Ref. cat.)	--	1 (Ref. cat.)	--
- At least one overweight	0.81 (0.56–1.17)	0.258	0.67 (0.43–1.06)	0.088
- At least one obese	0.68 (0.48–0.99)	0.041	0.47 (0.30–0.74)	0.001

BMI = body mass index. Improvement in Δ30BMI is defined as a post-pre decrease in Δ30BMI>= -1. BMI = body mass index. OR = odds ratio. CI = confidence interval. Ref. cat. = reference category.

[A] Δ30BMI refers to the difference between children's BMI and the age- and sex- specific BMI value corresponding to an adult BMI of 30 kg/m^2. Improvement in Δ30BMI is defined as a pre-post decrease in Δ30BMI>1. Improvement in weight class is defined as a change from a higher weight class to a lower one.

* Results obtained by two random-effect logistic regression models, using LHU as the cluster variable: the first predicting the improvement in Δ30BMI; and the second predicting the improvement in weight class. Both models included 1792 children and were not adjusted for mother's educational level and parents weight status, which were included into separate models (with all other covariates equal), because of 191 and 179 missing values, respectively. None of the other covariate estimates changed substantially after the inclusion of mother educational level and parents weight status.

[B] Adherence to recommended thresholds of international guidelines on lifestyle habits.
